# Performance Evaluation and Microstructure Characterization of Metakaolin-Based Geopolymer Containing Oil Palm Ash

**DOI:** 10.1155/2013/857586

**Published:** 2013-10-30

**Authors:** Abideng Hawa, Danupon Tonnayopas, Woraphot Prachasaree

**Affiliations:** ^1^Department of Civil Engineering, Prince of Songkla University, Songkhla 90112, Thailand; ^2^Department of Mining and Materials Engineering, Prince of Songkla University, Songkhla 90112, Thailand

## Abstract

This study reports on the microstructure, compressive strength, and drying shrinkage of metakaolin (MK) based geopolymers produced by partially replacing MK by oil palm ash (OPA). The OPA was used as raw material producing different molar ratios of SiO_2_/Al_2_O_3_ and CaO/SiO_2_. The geopolymer samples were cured at 80°C for 1, 2, or 4 hours and kept at ambient temperature until testing. The compressive strength was measured after 2, 6, and 24 hours and 7 and 28 days. The testing results revealed that the geopolymer with 5% OPA (SiO_2_ : Al_2_O_3_ = 2.88 : 1) gave the highest compressive strength. Scanning electron microscopy (SEM) indicated that the 5% OPA sample had a dense-compact matrix and less unreacted raw materials which contributed to the higher compressive strength. In the X-ray diffraction (XRD) patterns, the change of the crystalline phase after heat curing for 4 hours was easily detectable compared to the samples subjected to a shorter period of heat curing.

## 1. Introduction

The geopolymers are interesting in the fields of materials science and materials engineering. The geopolymer process is a chemical reaction between aluminosilicate materials and alkaline solutions under high curing temperature conditions. Generally, raw materials are prepared with a geopolymer binder consisting of fly ash and metakaolin (MK) containing SiO_2_ and Al_2_O_3_ which are the main chemical constituents. Geopolymers are binders that exhibit good physical and chemical properties and a wide range of potential applications [1]. However, several previous researches reported some of the limitations of geopolymer properties. Metakaolin based geopolymers show a relevant strength loss that makes them unsuitable for construction purposes [2]. There are efflorescence [3, 4] related problems with this materials, and recently, Turner and Collins [5] showed that sodium silicate “geopolymers” have almost the same carbon footprint as Portland cement.

Previous studies [6–8] have reported that different ratios of SiO_2_/Al_2_O_3_ influence the properties of the geopolymer binders. Generally, the geopolymer binder has been prepared using fly ash and metakaolin, in which the ratio of SiO_2_/Al_2_O_3_ varied within a range of 2 : 1 and 4 : 1. The effect of high calcium fly ash contents between 2.79 : 1 and 4.79 : 1 (SiO_2_/Al_2_O_3_) on the setting time and compressive strength of geopolymers was investigated in Chindaprasirt et al. [8]. The result showed that a higher compressive strength was achieved within a range of SiO_2_/Al_2_O_3_ ratios of 2.57 : 1 and 4.24 : 1. However, the current study focuses on SiO_2_/Al_2_O_3_ and CaO/SiO_2_ ratios.

van Jaarsveld et al. [9] used XRD and FT-IR techniques to characterize the fly ash obtained from different sources in order to gain a greater understanding of the effect of phase composition on the dissolution behavior, reactivity, and final physical and mechanical properties of fly ash-based geopolymers. The polymerization mechanism and the structure of the products were also investigated by Barbosa et al. [10] using XRD and FT-IR spectroscopy. A number of investigators [11, 12] have also studied the microstructure of geopolymers using SEM.

Oil palm ash (OPA) is a by-product of the use of palm kernels, palm fibers, and palm shells as biomass fuel in place of petroleum in electricity generation. Currently, OPA is disposed of in landfills, which has the potential to cause environmental problems for the industry and health risks for the public.

In the present study, geopolymers were prepared with OPA ratios of 0%, 5%, 10%, and 15% (hereafter referred to as Control, O5, O10, and O15, resp.), which produced differing ratios of SiO_2_/Al_2_O_3_ and CaO/SiO. They were prepared as hot mixtures using sodium silicate and sodium hydroxide as activators before being heat cured in an oven at 80°C for 1, 2, or 4 hours. The study aimed to analyze the effect of these parameters on the compressive strength and drying shrinkage using SEM, XRD, and FT-IR techniques. Measurements were taken after 2, 6, and 24 hours and 7 and 28 days.

## 2. Experimental Section

### 2.1. Materials

**Figure 1:**
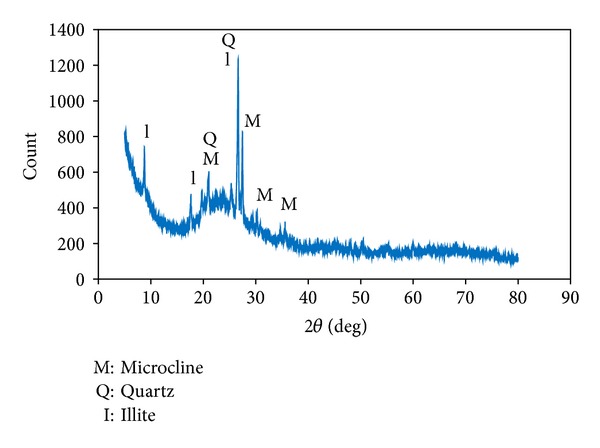
XRD pattern of MK.

The metakaolin (MK) used in this study was collected from Narathiwat province. It was calcined at 750°C for 2 hours and used as Si-Al cementitious material. The chemical composition of the MK was analyzed using X-ray fluorescence (XRF). The physical properties of the MK are listed in Table 1. Grinding the raw materials in a ball mill produced small particles.  Figure 1 depicts the XRD pattern of the MK. The MK showed an apparent amorphous phase (between 20 and 35°  2*θ*) in its structure with peaks for microcline, quartz, and illite.

**Figure 2:**
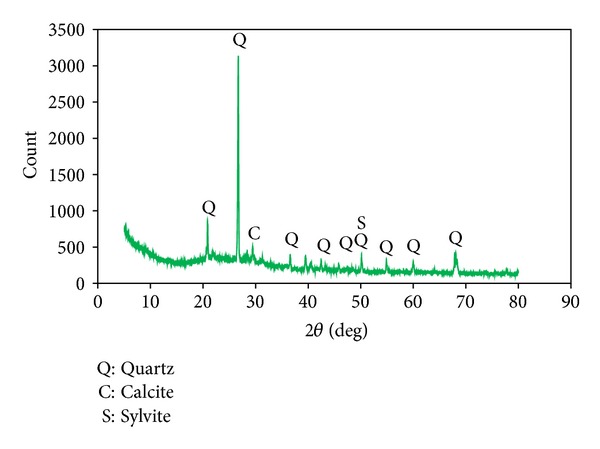
XRD pattern of OPA.

The OPA was obtained from a palm oil mill in Krabi province. It was sieved to remove any incompletely combusted fibers. The OPA was ground in a ball mill until the median particle size was approximately 19 *μ*m. The chemical composition and physical property of the OPA are shown in Table 1.  Figure 2 shows the X-ray diffractogram of the OPA, which demonstrates outstanding crystalline phase materials with obvious detectable quantities of crystalline quartz, calcite, and sylvite.

The activator used was a mixture of sodium hydroxide (NaOH) in flakes of 98% purity and sodium silicate (Na_2_SiO_3_). The sodium silicate solution had a composition by weight of 14.14% Na_2_O, 27.67% SiO_2_, and 56.28% H_2_O.

River sand was used as the fine aggregate component of the geopolymer mortars. The specific gravity of the river sand was 2.51, and the maximum size was 4.75 mm.

### 2.2. Mixture Proportion

The geopolymer mortars used in this study were prepared with MK, OPA, and the alkali activators sodium silicate and sodium hydroxide with a view to verify the viability of using geopolymer binders. All the samples contained a mass ratio of river sand : MK and OPA : alkaline activator : water of 3 : 1 : 0.83 : 0.45. The alkaline activator used was a mixture of sodium silicate and sodium hydroxide in a weight ratio of 2.5 : 1. The sodium silicate and sodium hydroxide were mixed in water and produced an exothermic temperature of 74 ± 2°C. Later, the river sand, MK, and OPA were added to the mixture which reduced the temperature to 48 ± 2°C. Samples of four different concentrations of OPA were prepared: 0% (Control), 5%, 10%, and 15%. The samples were quite sticky and fast setting and required some effort to be cast in acrylic molds. The samples of the four mixtures were then wrapped with a polyvinyl sheet to prevent any moisture loss and cured in an oven at 80°C for 1, 2, or 4 hours to produce all 12 different experimental conditions (as previously shown in Table 2), and after curing for 1, 2, or 4 hours, the samples were unwrapped, demolded, and allowed to further cure at an ambient temperature of 30 ± 2°C and 70 ± 5% relative humidity until the scheduled test date.

### 2.3. Test Procedure

After curing for 1, 2, or 4 hours at 80°C, the geopolymer mortars were removed from the acrylic molds and stored at ambient temperature. Compressive strength tests were conducted on cast specimens with dimensions of 50 × 50 × 50 mm. The samples were tested in accordance with the relevant ASTM C109/C109M [13] at ages of 2, 6, and 24 hours and 7 and 28 days. Drying shrinkage tests were performed during a period of up to 30 weeks using a length comparator in accordance with the relevant ASTM C490 [14].

A JMS-5800 LV model scanning electron microscope (JEOL, Japan) was used to identify the microstructure of the geopolymer mortars. Small scraps of the samples were tested using scanning electron microscopy.

Powder XRD was conducted using an X'Pert MPD X-ray diffractometer (PHILIPS, Netherlands) at angles from 5° to 80° (2*θ*) using the clay and rock 0.4 program. The MK, OPA, and geopolymer paste were characterized directly. XRD was conducted to identify the dominant crystalline phases and to detect the positions of the peaks.

FT-IR was performed on the geopolymer samples on an EQUINOX 55 spectrometer (Bruker, Germany) using the KBr pellet technique in 4000–400 cm^−1^ range.

## 3. Results and Discussion

### 3.1. Compressive Strength

#### 3.1.1. The Effect of the Ratio of SiO_2_/Al_2_O_3_

**Figure 3:**
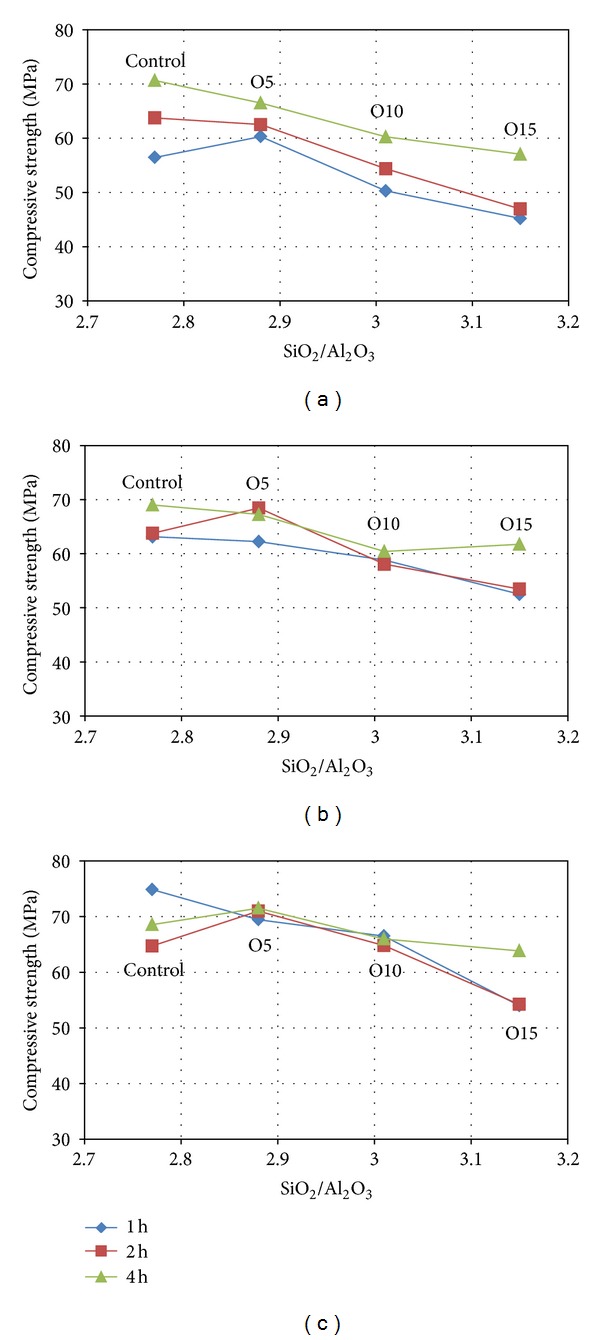
Compressive strength of geopolymer mortar with different SiO_2_/Al_2_O_3_ and heat curing time measured after (a) 2 hours, (b) 24 hours, and (c) 28 days.

**Figure 4:**
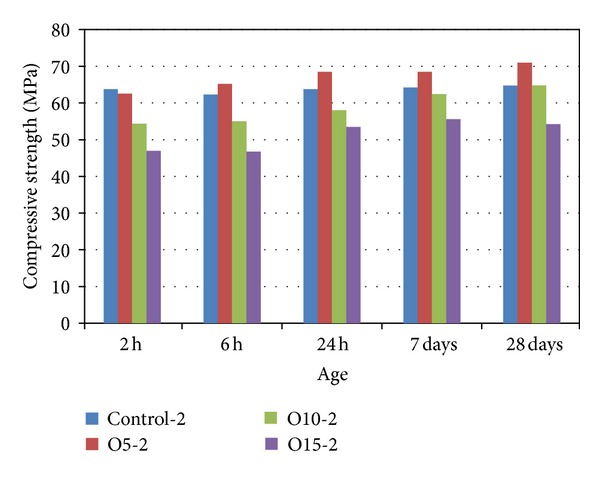
Compressive strength of geopolymer mortar heat cured for 2 hours.

The compressive strengths of the mortar after being cured at 80°C for 1, 2, or 4 hours are shown in Figures 3 and 4. It can be seen that for all the mixtures the compressive strength generally decreased with an increase in the proportion of SiO_2_ (molar ratio) after 2 and 24 hours at ambient temperature (see Figures 3(a) and 3(b), resp.).

The development of the compressive strength of the Control, O5, O10, and O15 samples heat cured for 1, 2, or 4 hours is shown in Figures 3(a), 3(b), and 3(c), respectively. It was observed that the initial ratio of SiO_2_/Al_2_O_3_ had a significant effect on the development of compressive strength in the geopolymer binder systems. It can be seen that after the shortest period of curing of 2 hours at ambient temperature (Figure 3(a)), the mixture with the highest Si content (O15) had the lowest compressive strength of all the combinations of Si content and period of heat curing, and increasing the period of heat curing while decreasing the Si content caused an increase in the measured compressive strength in all the samples.

Figure 3(a) illustrates the compressive strength measured after 2 hours at ambient temperature. It can be seen that the strength varied in the order Control > O5 > O10 > O15 (SiO_2_/Al_2_O_3_ ratios 2.77, 2.88, 3.01, and 3.15, resp.).

A somewhat similar trend in strength development was observed after 24 hours (see Figure 3(b)). However, the compressive strengths of O10 and O15 were higher than the comparative values after 2 hours (see Figure 3(a)). This behavior was due to the average particle sizes (*d*_50_: 6.31 *μ*m for MK against 19.31 *μ*m for OPA) being correlated to the specific surface area. The finer the particle size, the greater the surface area, which produces a more reactive material [9].

Figure 3(c) shows the variation in compressive strengths of the samples measured after 28 days. The most favorable SiO_2_/Al_2_O_3_ molar ratio for strength development in the geopolymer samples was found in O5 (SiO_2_/Al_2_O_3_ = 2.88), a trend similar to the findings of Chindaprasirt et al. [8] in respect of an increased alumina content (SiO_2_/Al_2_O_3_ up to 2.87) of high calcium fly ash-based geopolymer systems. However, in the present study, the compressive strengths of the samples containing only MK (SiO_2_/Al_2_O_3_ = 2.77) after 28 days were significantly higher for the samples cured for only 1 hour, which is consistent with the findings of Rovnaník [15].

The effect of the addition of OPA on compressive strength was slightly less for the control samples measured after 2 hours at ambient temperature, but beyond this age, the compressive strengths of the O5 samples were higher than those of the control samples after longer periods at ambient temperature. Therefore, in terms of compressive strength, the results suggest that the optimal OPA content is approximately 5%. It was also observed that the O5 samples continued to develop compressive strength to an age of 28 days. Because OPA has SiO_2_ as its main chemical component, the SiO_2_-to-Al_2_O_3_ ratio of the geopolymer product is improved by the addition of a small amount of OPA. However, adding larger amounts of OPA beyond the optimal amount decreases the compressive strength because the OPA also contains CaO. These results are consistent with those of previous studies [16] conducted on fly ash-based geopolymers.

#### 3.1.2. The Effect of Heat Curing

The compressive strengths of the geopolymer mortars as a function of heat curing and the amount of OPA as a replacement for MK are illustrated in Figure 3. For all the mixtures, longer heat curing was found to accelerate the development of compressive strength after 2 hours at ambient temperature more than a shorter period of heat curing. Longer heat curing may accelerate the degree of geopolymerization because of the formation of mineral phases. However, as Figure 3 shows, the compressive strengths of the control samples containing only MK after 28 days at ambient temperature were significantly higher for the samples cured for only 1 hour, which is consistent with the findings of Rovnaník [15]. However, for the O5 and O10 samples cured for 1, 2, or 4 hours containing 5% and 10% OPA, the compressive strength values at 24 hours and 28 days were similar with curing periods of 1, 2, or 4 hours (as shown in Figure 3), while the O15 samples containing 15% OPA, heat cured for 4 hours had the highest compressive strengths. The influence of heat curing on the compressive strengths of samples cured for 1 and 2 hours was nearly the same after all periods at ambient temperature, as illustrated in Figure 3.

### 3.2. Drying Shrinkage

**Figure 5:**
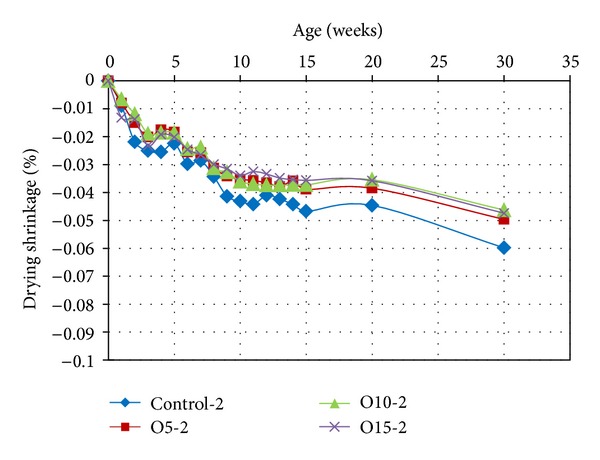
Drying shrinkage of geopolymer mortars containing OPA, heat cured for 2 hours.

**Figure 6:**
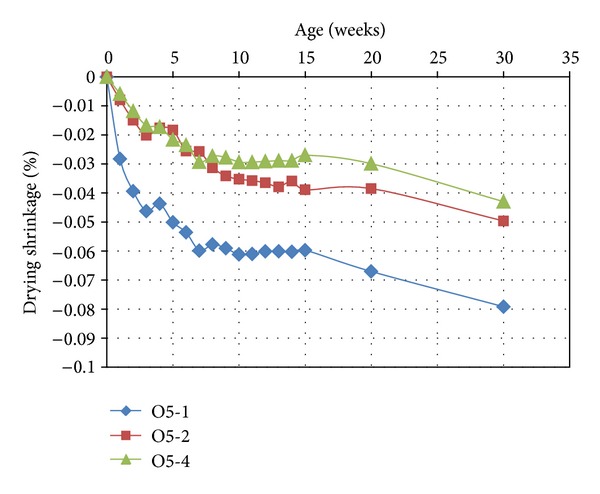
Drying shrinkage of geopolymer mortar containing 5% OPA.

The effect of the partial replacement of MK with OPA on the drying shrinkage of the geopolymer mortars is presented in Figures 5 and 6. The overall result indicated that the drying shrinkage was very low. The drying shrinkages for different proportions of OPA are illustrated in Figure 5. A comparison of the measurements of the control samples heat cured for 2 hours (Control-2) shows that the drying shrinkage values decreased over time. In addition, the decrease in drying shrinkage was inversely proportional to the increase in the OPA content. This lower drying shrinkage is due to the lower fineness of the geopolymer mortar with higher OPA content. This is similar to the trend of drying shrinkage reduction reported in Chareera [17]. It has previously been confirmed that geopolymers with fine-sized calcined kaolin particles produce high shrinkage [18]. This phenomenon is due to fine particles having a larger geopolymerization reaction surface area, and if they are packed inadequately into a slurry system, they will produce high shrinkage.

The drying shrinkage of geopolymer mortar containing OPA cured for 1, 2, or 4 hours was similar, producing decreased drying shrinkage with longer curing time at elevated temperature. For example, the O5 sample cured for 2 and 4 hours had similar shrinkage values at all ages, with drying shrinkage decreasing up to an age of 8 weeks, as shown in Figure 6. Thereafter, the drying shrinkage values decreased slowly. However, the drying shrinkage values in the first 8 weeks for the samples cured for 4 hours were less than those of the samples cured for 2 hours. On the other hand, for the geopolymer mortar cured for 1 hour, the drying shrinkage values were much higher at all ages than those for the samples cured for 2 or 4 hours. This was because longer curing times at elevated temperature result in a loss of water due to treatment heating during geopolymerization.

**Figure 7:**
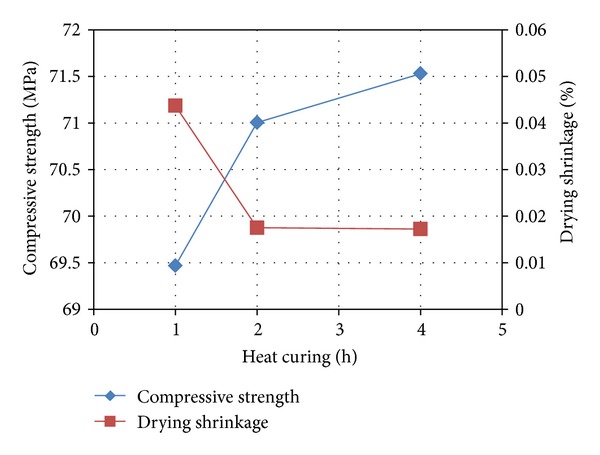
Effect of heat curing on compressive strength and drying shrinkage of samples with 5% OPA at 28 days.

The effect of heat curing on compressive strength and drying shrinkage is depicted in Figure 7. It is evident that while the compressive strength increases with greater periods of heat curing, the drying shrinkage decreases especially between periods of 1 and 2 hours. The optimum period of heat curing is 2 hours which produces geopolymers with the lowest drying shrinkage and reasonably high strength.

### 3.3. Microstructure Characterization

#### 3.3.1. Scanning Electron Microscope (SEM)

**Figure 8:**
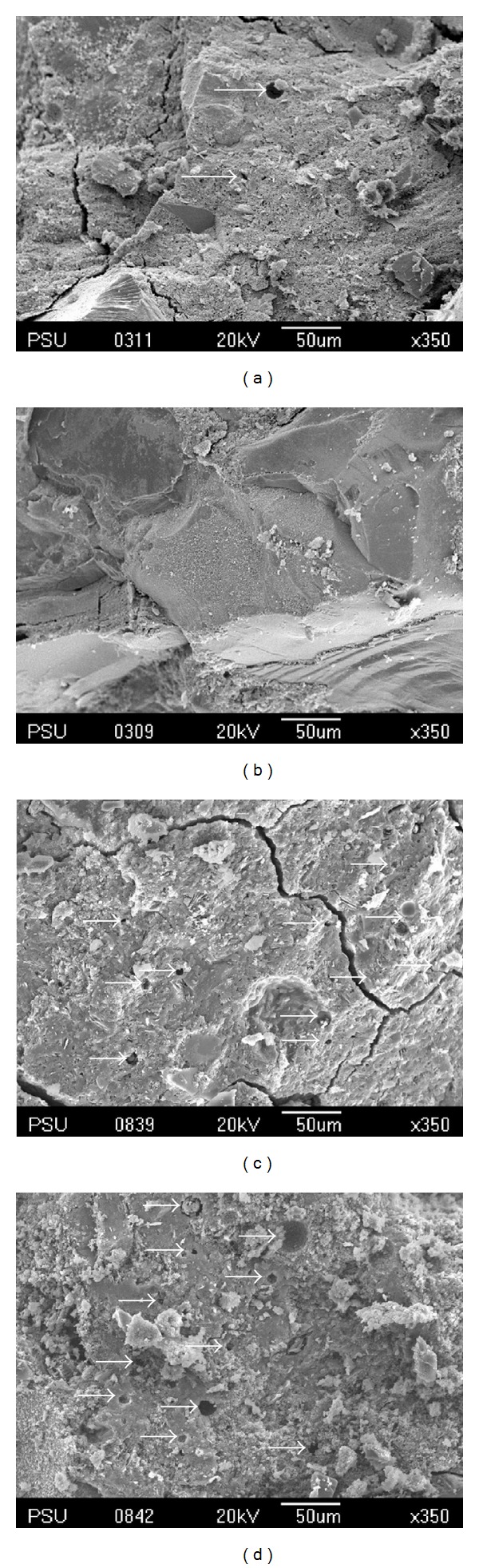
SEM micrograph of geopolymer mortar; (a) Control-2, (b) O5-2, (c) O10-2, and (d) O15-2.

The microstructure of the MK-based geopolymers with different OPA contents was observed by SEM, and the results are shown in Figure 8. The comparison of the SEM pictures of the sample matrices revealed that some raw materials, which had not reacted, were partially coated with flakes that formed on the crust of the samples. It was notable that the Control-2, O10-2, and O15-2 matrices were not homogeneous and contained small pores. The O15-2 sample in particular had many flakes and the largest pores (see Figure 8(d)), whereas in the O5-2 matrix, a lower proportion of unreacted raw materials was detected in the samples (see Figure 8(b)). It was also found that the O5-2 samples had higher homogeneity and the lowest number of pores in comparison to the other samples with the least unreacted MK and OPA from the alkaline activator. This sample also produced the highest compressive strength of 70 MPa after a period of 28 days. This suggests that the dissolution of the aluminosilicate in the geopolymerization process in the O5-2 sample produced the highest compressive strength. The pores in the geopolymer matrices which lead to the lower compressive strength are shown in Figures 8(a), 8(b), and 8(c) by arrows.

**Figure 9:**
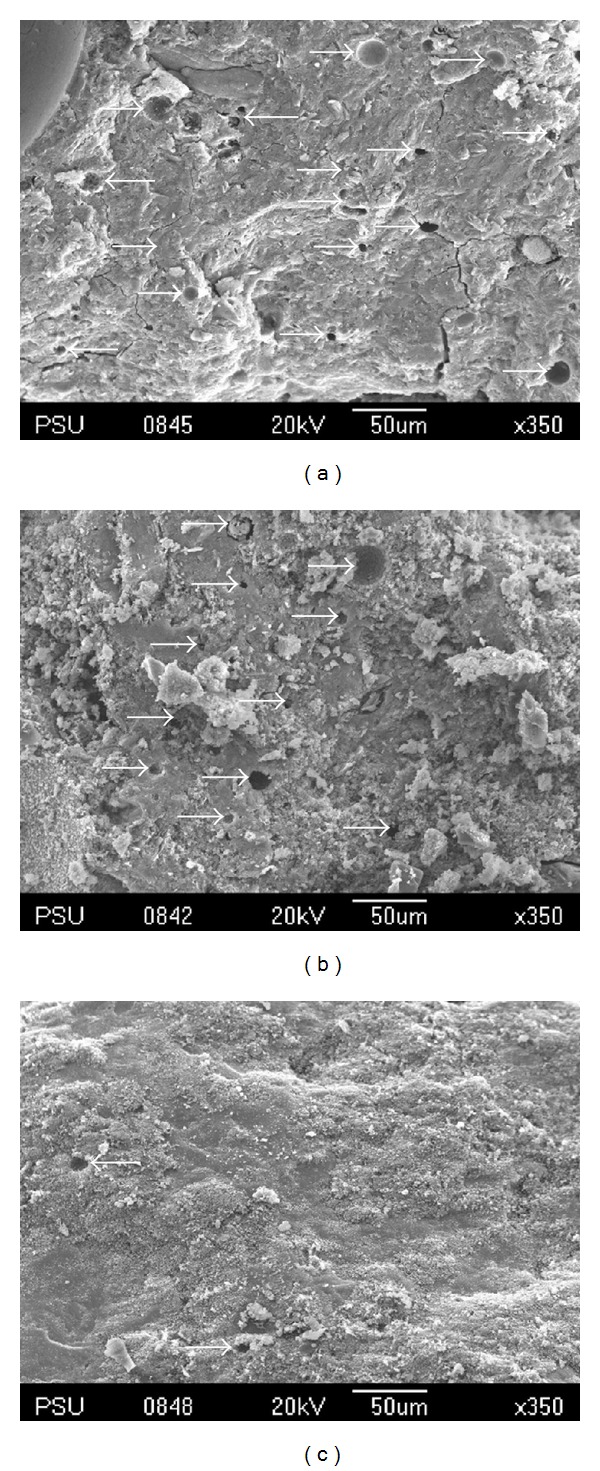
SEM micrograph of geopolymer mortar; (a) O15-1, (b) O15-2, and (c) O15-4.

As the heat curing was increased from 1 to 4 hours, the microstructure of the MK-based geopolymer containing OPA demonstrated different proportions of unreacted raw materials as can be seen in Figures 9(a) and 9(b). It was observed that the O15-1 and O15-2 samples were covered in flakes of unreacted raw materials that had formed on their crust and they also contained many pores in the matrix as illustrated in the figures. by arrows. Nevertheless, in the O15-4 sample, (Figure 9(c)) it was observed that the matrix was homogeneous with a dense-compact microstructure and a lower proportion of unreacted raw materials. This is consistent with the finding of higher compressive strength in Figure 3(c).

#### 3.3.2. X-Ray Diffraction (XRD)

**Figure 10:**
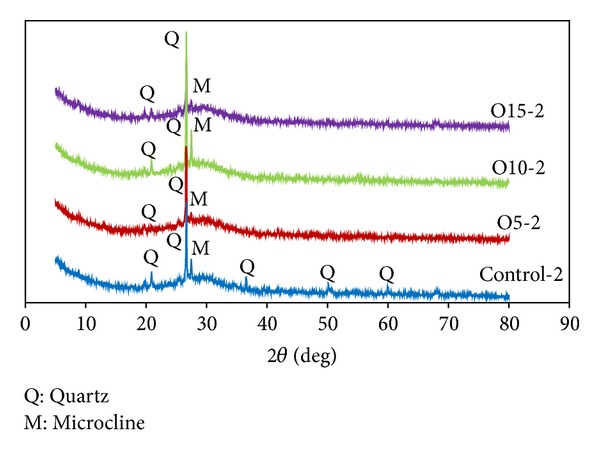
XRD patterns of geopolymers containing OPA, heat cured for 2 hours.

The results of the XRD of the MK-based geopolymers containing 5, 10, and 15% OPA, heat cured for 2 hours are shown in Figure 10. The full mixture showed a characteristic high background between 15° and 35° 2*θ* with a decrease in the crystalline peaks associated with the initial materials. The samples had a similar diffraction pattern and did not demonstrate any significant change in the degree to which they were amorphous and crystalline from the control sample. The peaks of quartz, microcline, and illite from the MK and of quartz, calcite, and sylvite from the OPA were observed to have almost disappeared, indicating a degree of geopolymerization. However, the peak of the quartz content was noted to be around 27° 2*θ* due to remaining prominent quartz in both MK and OPA.

**Figure 11:**
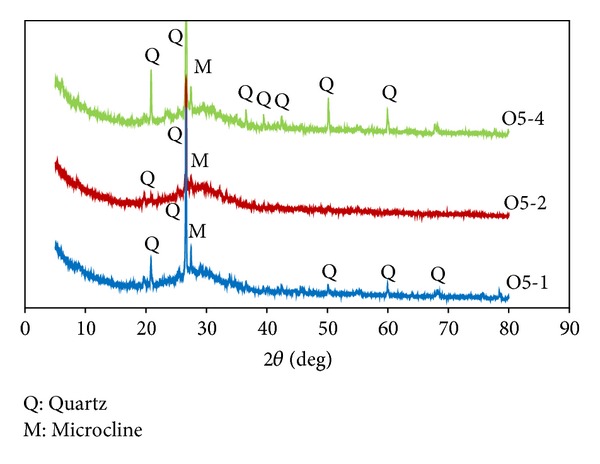
XRD pattern of geopolymer containing 5% OPA.

Figure 11 shows the XRD patterns for sample O5 with various heat curing at 1, 2, and 4 hours. The three samples had a similar pattern being mostly amorphous with some crystalline peaks. The XRD pattern for the 4-hour sample shows that the apparent quartz content was detected most obviously at approximately 21°, 27°, 50°, and 60° 2*θ* compared to the samples heat cured for 1 and 2 hours. This indicates that crystalline phases were detected in the geopolymer samples and that heat curing at 4 hours produced the highest amount of crystallinity and had a higher compressive strength. Álvarez-Ayuso et al. [19] reported that fly ash-based geopolymers with increased crystallinity exhibited increased compressive strength. It has also been found that fly ash-based geopolymer with 12 M NaOH showed the highest compressive strength, and XRD results showed that the intensity of the crystallinity was easily detectable and contributed to the highest compressive strength [20].

#### 3.3.3. Fourier Transform Infrared Spectroscopy (FT-IR)

**Figure 12:**
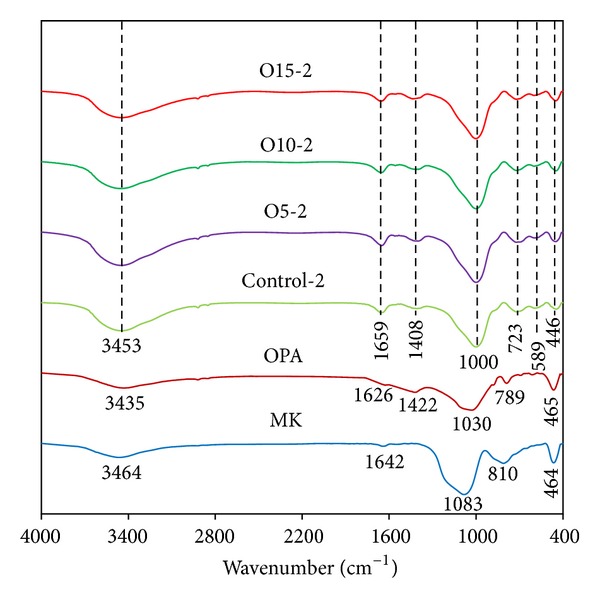
FT-IR spectra of raw materials and the geopolymer samples heat cured for 2 hours.

Figure 12 shows the FT-IR spectra for the Control-2, O5-2, O10-2, and O15-2 samples with those of MK and OPA for purposes of comparison. The MK spectrum contains wide bands at approximately 1083 and 464 cm^−1^, reflecting the Si–O vibrations. These major bands are observed at frequencies near to these reported in the literature for this compound [9, 21]. There is also a band at 810 cm^−1^ corresponding to Al–O. For the OPA spectra, the stretching of the Si–O groups alternately bound to the Al–O bonds produces a signal at 1030 cm^−1^ and a band around 789 cm^−1^ indicative of the Al–O or Si–O–Al groups. These major bands have also been reported in the literature [22].

The FT-IR spectra of the Control-2 sample (see Figure 12) indicate major bands at approximately 3453, 1659, 1408, 1000, 723, 589, and 446 cm^−1^. Those at 3453 and 1659 cm^−1^ are formed by the O–H stretching vibration and the H–O–H bending vibration, respectively, and these have been previously noted in hydroxyl groups [9, 23]. The main binder system vibration band at approximately 1000 cm^−1^ is attributable to the asymmetric stretching mode of the Si–O–Al bond in the reaction products, and this vibration band has also been found in previous research [24]. The main bands in the geopolymers are only a little different from those in the FT-IR spectra of the MK between 450 and 1200 cm^−1^. It has been previously reported that some raw material is retained in the geopolymerization products [25]. The results of the present study suggest that the composition of the aluminosilicate was formed by the geopolymerization of the MK and/or the OPA and the alkaline activator produces slightly different FT-IR patterns. The effect of the OPA ratio on the nanostructure as shown by the FT-IR spectra in Figure 12 is rather limited. The trend of the FT-IR spectra in previous research has been similarly adopted [26] where it has been reported that difference in the *w*/*b* ratio in the gel nanostructure of the fly ash based geopolymer as displayed by the FT-IR spectra is rather limited.

**Figure 13:**
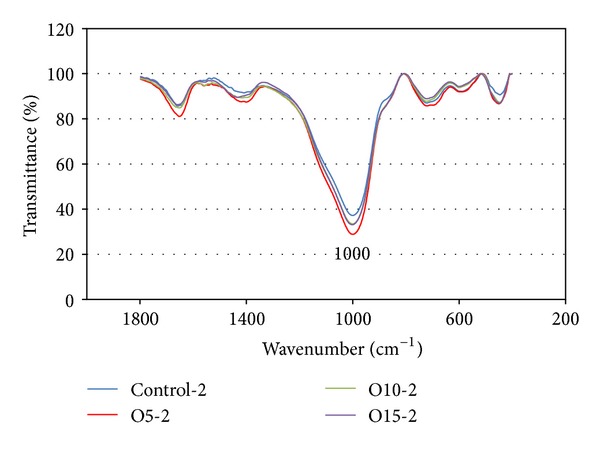
FT-IR spectra of the geopolymer samples.

It was observed that the main binder system vibration band occurred at approximately 1000 cm^−1^ which can be attributed to the asymmetric stretching mode of the Si–O–Al bond, as shown in Figure 13. However, the compressive strength of the geopolymer mortars produces a trend of lower transmittance (high absorption), which reflects the higher strength of the geopolymer as detected in the FT-IR test.

## 4. Conclusions

The effect of the partial replacement of MK by OPA, different periods of heat curing on the microstructure, and compressive strength of a MK-based geopolymer were investigated, and the following conclusions may be deduced.

- The O5 sample (SiO_2_ : Al_2_O_3_ = 2.88 : 1) produced the highest compressive strength. The MK-based geopolymer, heat cured for 4 hours, had the highest compressive strength of all. The XRD results showed that the intensity of the crystalline phase after heat curing for 4 hours was easily detectable and contributed to the higher compressive strength more than the samples heat cured for 1 and 2 hours.
- The CaO content (CaO/SiO_2_ = 0.04) was hostile in the geopolymer produced, especially in relation to the early strength measurement after 2 hours at ambient temperature and heat curing for 1 and 2 hours. However, heat curing for 4 hours produced higher strength.
- The alkali activation of MK with sodium silicate and sodium hydroxide solutions produced materials with high early compressive strength, when prepared in a hot mixture.
- The addition of OPA, from 5% to 15%, had the effect of decreasing the drying shrinkage of the geopolymer mortars.
- Long heat curing times also decreased drying shrinkage, probably due to the well-developed strength.

The increased compressive strength was attributable to the structure of the geopolymer samples which had a dense-compact matrix and contained less unreacted raw materials. Further, a higher reaction of Si–Al in the geopolymerization process produced aluminosilicate, and in addition, the preparation of the geopolymers in a hot mixture in this study may have also contributed to the compressive strength. However, different MKs from other locations may need different ratios and particle sizes to achieve high compressive strength.

## Figures and Tables

**Table 1:** Chemical composition (wt.%) and physical property of MK and OPA.

Element	MK	OPA
SiO_2_	50.30	38.37
Al_2_O_3_	41.02	1.48
CaO	0.33	13.84
Fe_2_O_3_	1.05	3.01
K_2_O	4.08	14.09
TiO_2_	1.50	0.21
MgO	—	3.00
Other	—	5.57
LOI	1.72	20.43

Specific surface area (m^2^/g) BET	13.61	13.06

*d*_10_ (*μ*m)	1.352	4.321
*d*_50_ (*μ*m)	6.308	19.305
*d*_90_ (*μ*m)	88.803	100.109

**Table 2:** Mix proportions of geopolymer samples.

Sample name	MK (by weight)	OPA (by weight)	CaO/SiO_2_ (molar ratio)	SiO_2_/Al_2_O_3_ (molar ratio)	Heat curing (h)
Control-1	100	—	0.01	2.77	1
O5-1	95	5	0.02	2.88	
O10-1	90	10	0.03	3.01	
O15-1	85	15	0.04	3.15	

Control-2	100	—	0.01	2.77	2
O5-2	95	5	0.02	2.88	
O10-2	90	10	0.03	3.01	
O15-2	85	15	0.04	3.15	

Control-4	100	—	0.01	2.77	4
O5-4	95	5	0.02	2.88	
O10-4	90	10	0.03	3.01	
O15-4	85	15	0.04	3.15	
